# Do different survey methods affect physicians’ stated work preferences? Findings from a discrete choice experiment in Eastern China

**DOI:** 10.3389/fsoc.2024.1474795

**Published:** 2024-12-20

**Authors:** Lizhu Wang, Dan Hu, Jing Zhu

**Affiliations:** ^1^School of Health Policy and Management, Nanjing Medical University, Nanjing, China; ^2^Laboratory for Digital Intelligence & Health Governance, Nanjing Medical University, Nanjing, China; ^3^Division of Medical Affairs, The Affiliated Jiangning Hospital of Nanjing Medical University, Nanjing, China

**Keywords:** discrete choice experiment, human resource, workforce performance, work preferences, survey methods

## Abstract

**Objectives:**

This study aimed to examine the impact of online and offline survey methods on the participation of physicians in discrete choice experiment (DCE) surveys in Eastern China and explore their attitudes towards primary healthcare work.

**Methods:**

The study involved active doctors practicing at secondary or county-level general hospitals in Eastern China, who completed a DCE questionnaire either online or offline. A mixed logit model was used to analyze the data, considering the relative importance of various job attributes.

**Results:**

This study found that online surveys save costs and offline surveys help increase the response rate for questionnaires. The validity rate for the completed questionnaires was high (>90%) across both research methods. A mixed logit model simulation analysis revealed that compensation packages were the dominant influence on doctors’ choices. The online survey showed that doctors were more likely to choose to work in village health centers if their salary was flat (*β* = 1.330), while the offline survey showed that doctors were also more likely to choose village health centers when their salary was increased by 10% (*β* = 1.095). Work organization and public recognition also had a significant effect on doctors’ primary job choices (*p* < 0.05).

**Conclusion:**

The study concluded that remuneration, work organization, and public recognition are key factors affecting physicians’ willingness to work in primary healthcare settings. For respondents with higher education and cognitive abilities, online surveys are recommended for DCE research.

## 1 Background

According to World Health Organization recommendations, 70–80% of basic diseases can be effectively treated in primary healthcare institutions (Rivo, 2009). Thus, an institution’s capacity to deliver primary healthcare services is a critical influence on residents’ choice of healthcare facilities, leading to cost savings and enhanced efficiency of healthcare service utilization. Augmenting the supply of human resources is a crucial consideration in the provision of primary health care, with quality healthcare personnel being the fundamental force behind the optimization of basic healthcare service delivery. However, global primary healthcare systems are currently grappling with the challenges presented by a diminishing labor supply coupled with escalating demand for primary care physicians (Liu et al., 2017). The scarcity of adequate primary health care personnel has emerged as a significant bottleneck that is hindering the evolution of China’s primary health care system.

Although China has made significant progress in strengthening its primary healthcare system, it is still faced with a shortage of high-quality primary healthcare physicians. The issue of a “reverse rate of health services” is particularly pronounced in China about disease burden, accessibility of health resources, and utilization of health services. The term “reverse rate of health services” refers to the primary healthcare services that are experiencing the highest level of demand but are also faced with the smallest allocation of healthcare personnel. The level of health worker allocation in China is currently significantly lower than that that exists in the European Union (Islek and Sahin, 2023). According to the China Health Statistics Yearbook (National Health Commission of the People’s Republic of China, 2019), the number of rural primary doctors and health workers in China decreased from 795,500 in 2020 to 696,700 in 2021, representing a decline of 12.42%. To address it, China has primarily focused on training general practitioners to fill the gaps, and on integrating high-quality human resources into grassroots development through medical associations. However, these measures have demonstrated limited efficacy thus far. Given they function as the core personnel of the primary healthcare workforce, the training model for general practitioners emphasizes the “5 + 3” training in higher medical schools alongside on-the-job transfer and training. This approach presents challenges in terms of training the required number of qualified general practitioners within a short period. Higher-level hospitals within medical unions also notably dispatch professional and technical talent to primary medical institutions to provide clinical teaching and business guidance. However, the transient nature of this talent deployment means that it has a limited impact in terms of enhancing the capacity of primary healthcare services and fails to substantially improve the internal structure of grassroots teams. A research recommended that China should focus on improving the quality of primary healthcare providers by adjusting the incentives and system of governance provided (Soekhai et al., 2019). The operational vitality of primary healthcare organizations can be stimulated by tapping into the job preferences of healthcare workers at the grassroots level and establishing effective incentive mechanisms that can attract qualified doctors to participate. This would improve the capacity of primary health services, enhance the residents’ satisfaction levels, encourage patients to gradually return to grassroots facilities, and reduce unnecessary health costs.

The physicians’ stated work preferences challenges faced by global primary healthcare systems are complex and multifaceted. Current scholars have combined many theories to address the work preferences. Some scholars have combined prospect theory to argue that workers may pay a greater psychological cost from the negative aspects of their jobs (e.g., burnout, low pay, lack of support) than they do from the rewarding aspects (Maslach and Leiter, 2016). Some scholars have argued, in conjunction with expected utility theory, that the utility that PCPs derive from their work is influenced by a variety of factors, such as financial compensation, job security, work-life balance, opportunities for career advancement, and the potential to make a difference in the lives of their patients (Kim et al., 2023). A study incorporating social appropriateness theory argues that rarely do physicians choose to stay in primary care due to social pressures out of a sense of duty or obligation to the community, especially in medically underserved areas (Bhatnagar et al., 2017). Some scholars, in conjunction with Maslow’s hierarchy of needs theory, argue that the basic needs of healthcare workers, such as fair wages, adequate working hours, and safe working conditions, are fundamental. If these needs are not met, healthcare workers are less likely to be motivated and may leave their positions (Blaauw et al., 2013). Thus, these theories can provide a nuanced understanding of the factors influencing the availability and retention of primary healthcare workers.

Discrete choice experiment (DCE), a crucial measurement tool, has been extensively utilized to elucidate individual preferences and decision-making processes in various choice scenarios. Since the late 1990s, DCE has been progressively adopted in the field of health human resources, particularly in analyzing the career choices of medical personnel, highlighting its unique benefits (Mandeville et al., 2014). DCE effectively quantifies the impact of different decision factors on the job choices of doctors and other medical personnel by creating choice situations with varied attribute combinations to mimic the individual decision-making process (Soekhai et al., 2019). With the rapid advancement of information technology, especially in the wake of the COVID-19 pandemic, online DCE, leveraging its ease of use, lower costs, and broad reach, has emerged as a survey instrument increasingly utilized globally in recent years. However, the implications of online versus offline surveys in capturing physicians’ choice preferences, particularly how it influences the accuracy and representativeness of survey outcomes, remains an area worthy of further investigation. Furthermore, DCE studies predominantly concentrate on the job choices of medical students, nurses, and healthcare workers in tertiary hospitals, with fewer studies focusing on healthcare workers in secondary or district hospitals. Research indicates that physicians’ job choices are influenced by a myriad of factors, including but not limited to remuneration packages, career development opportunities, work environment, and social benefits (Rao et al., 2013; Park and Ko, 2016; Rana et al., 2016; Saran et al., 2020; Law et al., 2021; Ajisegiri et al., 2022). For instance, in developed countries, remuneration and the desirability of the workplace often serve as the primary determinants of job choice for physicians (Park and Ko, 2016; Li et al., 2023), whereas in developing countries like Ethiopia and India, factors such as continuing education opportunities, promotion training, and fixed salaries are particularly significant (Abdel-All et al., 2019; Oladeji et al., 2022). In China, remuneration packages are particularly pivotal, especially for medical students and nursing staff from rural backgrounds, where salary level often plays a decisive role in the decision to pursue careers in primary care organizations (Bao and Huang, 2021; Bao et al., 2023). Thus, enhancing salary levels is viewed as a viable strategy to improve the appeal of primary care organizations. Bianzhi in China signifies a stable form of employment, thereby exerting a pronounced influence on doctors’ work preferences (Liu et al., 2022). According to Maslow’s hierarchy of needs theory, doctors’ sense of professional value and social status also influence their career choices (Manahan et al., 2009; Manda et al., 2023). Specifically in China, physicians continue to place a relatively high value on public recognition (Poh et al., 2022).

Although the current body of research has demonstrated that physicians’ decisions regarding primary care work are influenced by a variety of factors including personal, career development, and economic considerations, there remains a notable gap in the literature concerning the primary work preferences of active physicians in secondary hospitals in China. Furthermore, there is also a notable dearth of research addressing the impact of online versus offline survey methods on the outcome of DCE surveys. Thus, in the interest of filling this gap, the principal objective of this study was to examine the impact of online and offline survey modes on physicians’ participation in DCE surveys within secondary or county-level general hospitals in Eastern China. This study aims to explore the relative importance and probability of this population’s preference for engaging in primary care work by combining and presenting a variety of different options. Firstly, this study engaged in a comprehensive descriptive statistical analysis using both online and offline survey methods. The terms of this analysis encompassed the cost, questionnaire quality, and the reasons why physicians declared themselves reluctant to engage in primary care provision. Subsequently, a mixed logit model analysis was employed to calculate the heterogeneity, importance, and probability of the specific job attribute preferences of physicians in secondary hospitals under diverse program combinations. It is hoped that this study will serve as a foundational resource for guiding the selection of methodologies for subsequent DCE surveys of physicians and offer policymakers more nuanced and specific recommendations for incentive programs.

## 2 Methods

In this study, we opted to employ DCE to gauge the preferences and factors influencing physicians’ decisions regarding primary care work in secondary or county-level general hospitals in Eastern China. The use of the DCE method enabled the simultaneous assessment of multiple attributes. Participants were tasked with selecting from various scenarios, each comprising a unique combination of job attributes.

### 2.1 DCE design

After a comprehensive literature review coupled with focus group interviews, importance ranking, and expert consultations, the research team finalized six job attributes with 13 levels.

As presented in Table 1, these influencing factors encompassed work organization, remuneration packages, title promotion, type of employment, weekly working hours, and level of recognition and respect from residents. Notably, the attribute related to work establishment included a unique feature specific to China—“bianzhi.” It signifies that the job undertaken guarantees lifetime employment by the government and that the employee cannot be ordinarily dismissed by their employer (Liu et al., 2022).

**Table 1:** DCE design.

No.	Attribute	Description	Level
1	Working organization	Types of primary health care organizations that may be served	Village health office Township health center Community health service center
2	Remuneration package	The extent to which annual income has changed from its current level. Annual income includes salary, bonuses and various benefits and allowances	20% lower than the current salary The same as the current salary 10% higher than the current salary
3	Title promotion	Changes in the time required to move up one level in the title grade compared to the situation in the current hospital	Consistent with the hospital’s promotion year One-year advance promotion
4	Types of employment	Types of hiring for personnel management positions for employees in primary care structures	No bianzhi Recorded Bianzhi
5	Weekly working hours	Combination of acceptable weekly daily workload and overtime hours	60 h and above 40 h and below 40–60 h
6	Recognized and respected by residents	Can be recognized and respected by residents, personal value, sense of honor and social influence, etc. are reflected or enhanced	Not recognized and respected Generally relatively recognized and respected

These variables constituted the essential components of the experimental design. Specifically, one attribute was configured at the two levels, and five attributes were delineated at three levels. The full combination of attribute levels yielded 486 (2 × 3^5^) distinct attribute combinations. To streamline and facilitate the experiment, 12 choice sets were constructed using the D-efficient design provided by the Ngene DCE design software. To enhance the participants’ engagement and comprehension, 12 choice sets were evenly distributed across the two different questionnaire versions. In addition, a validation option set was established within each questionnaire set. Consequently, each participant was presented with queries pertaining to seven pairs of scenarios. The questionnaires were meticulously tailored and specifically designed for secondary care physicians. Examples of the option sets are presented in Table 2.

**Table 2:** Examples of selection sets (translated from the original Chinese version into English).

Job attributes	Job 1	Job 2
Work organization	Township health center	Community health service center (station)
Salary and benefits	Same as the current salary	20% lower than the current salary
Title promotion	In line with the promotion period in hospitals	Promotion 1 year in advance
Type of employment	Record system	No establishment
Weekly working hours	40–60 h	40 h or less
Recognition and respect from residents	No recognition and respect	General
Which would you prefer, “Job 1” or “Job 2”? Please tick ✓	□	□

### 2.2 Data collection for both research methods

This cross-sectional study aimed to explore the willingness and preferences of doctors in secondary or county-level general hospitals in Eastern China regarding employment in primary healthcare organizations. In July 2023, in collaboration with the Yancheng Health Commission, this study employed a dual methodology, utilizing electronic questionnaires through online software, and distributing paper questionnaires face-to-face via research investigators. For the offline survey, three districts—Jianhu, Tinghu, and Dongtai—were randomly chosen as the designated sample areas in Yancheng City. These three areas have a resident population of 600,000–800,000 people. Over the course of 2 days, the entire cohort for the offline survey included doctors on duty in secondary hospitals within these areas. Over the course of 3 days, doctors who were not on duty on that given day were surveyed using online electronic questionnaires. In total, 298 electronic questionnaires and 175 paper questionnaires were collected. There were 88 online data and 51 paper data from Jianhu District, 108 online data and 70 paper data from Tinghu District, and 92 online data and 54 paper data from Dongtai District.

### 2.3 Data analysis

First, this study undertook a comprehensive examination of descriptive statistics pertaining to the identity category, sensitivity category, satisfaction evaluation questions, and reasons for the reluctance of respondent doctors to work in primary healthcare organizations.

Second, the DCE data were analyzed using a mixed logit model. Because of its ability to accommodate a diverse range of mixed distributions, including discrete and continuous distributions. This model’s high level of flexibility allows it to approximate any random utility model (Yuan et al., 2022). This attribute has seen it be widely employed across a range of DCE studies focusing on human capital (Li et al., 2014; Liu et al., 2019).

In this study, a mixed logit model was constructed using survey mothods as the categorization criterion as follows:


$$\begin{array}{l} \mathrm{Unit}=\beta_{1}{\mathrm{Organization}}_{\mathrm{Township}}+\beta_{2}{\mathrm{Organization}}_{\mathrm{Community}}\\ +\beta_{3}{\mathrm{Income}}_{\mathrm{same}}+\beta_{4}{\mathrm{Income}}_{10\%\mathrm{higher}}\\ +\beta_{5}{\mathrm{Promotion}}_{\mathrm{one}\;\mathrm{year ahead}}+\beta_{6}{\mathrm{Employment}}_{\mathrm{Record system}}\\ +\beta_{7}{\mathrm{Employment}}_{\mathrm{bianzhi}}+\beta_{8}{\mathrm{Working hours}}_{40-60\;\mathrm{hours}}\\ +\beta_{9}{\mathrm{Working hours}}_{40\;\mathrm{hours below}}+\beta_{10}{\mathrm{Recognition}}_{\mathrm{Average}}\\ +\beta_{11}{\mathrm{Recognition}}_{\mathrm{More}}+\mathrm{\varepsilon nit} \end{array}$$

Subsequently, the relative importance (RI) of each attribute was computed by summing the utility difference between the horizontal maximum and minimum regression coefficients within a specific attribute and the utility difference across the horizontal regression coefficients within all attributes of the mixed logit model (Determann et al., 2016). It was modeled as follows:


$${RI}_{k}=\frac{\max\beta_{k}-\min\beta_{k}}{\sum_{k=1}^{k}\left(\max\beta_{k}-\min\beta_{k}\right)}$$

Finally, this study developed a parametric probability model for scenario prediction analysis to provide an intuitive measurement of the effects of policy changes. It was modeled as follows:


$$P_{i}=\frac{e^{\beta_{\mathrm{Option}\;i}}}{\sum e^{\beta_{\mathrm{Option}\;i*\mathrm{Option}\;j}}}$$

The entire analytical process was executed through STATA 18.0.

## 3 Results

### 3.1 Comparison of the cost and quality of the two survey methods

As shown in Table 3, in the perspective of time-cost, the distribution of questionnaires took 9 days to administer online and 2 days to administer offline. On average, each electronic questionnaire required 9.3 min to complete, while each paper questionnaire took 10–12 min. In terms of monetary costs, the offline method incurred expenses totaling 3,780 RMB. These costs encompassed transportation, accommodation, catering, and the printing of paper questionnaires for the 10 investigators. The online method incurred expenses 0 RMB.

**Table 3:** Basic information statistics of the two research methods.

	Volume		Online	Offline
Cost	Number of days for data collection		9	2
	Time to complete each questionnaire (minutes)		9.3	10–12
	Money invested (CNY)		0	3,780
	Number of investigators		1	10
Quality	Number of districts covered		5	3
	Number of hospitals covered		5	3
	Original sample size		298	175
	Excluded sample size	Validation option set selection error	10	2
		Filling time <2 min	11	0
		Vacant values ≥10%	0	5
	Valid sample size		277	168
	Valid sample rate		92.95%	96.00%

In the perspective of lobar-cost, participants in the offline survey completed a questionnaire with individual explanations provided by 10 investigators. Conversely, in the online survey, a single questionnaire designer created a survey using the Questionnaire Star software. Links to the e-questionnaire were disseminated through internal WeChat (a Chinese instant messaging application) groups across all levels of the regional health system. The e-questionnaire also provided background information on the six job attributes.

An assessment of questionnaire quality revealed that after excluding invalid questionnaires with incorrectly selected validation options, completion times below 30 s, and questionnaire vacancies equal to or exceeding 10%, the total sample size of the online questionnaire exceeded that of the offline questionnaire. However, the sample validity of the offline paper questionnaire also surpassed that of the online e-questionnaire.

### 3.2 Description of the study sample

The questionnaire used in this study comprised four distinct categories. The initial category encompassed identity characteristics, the second involved life satisfaction, the third pertained to sensitive topics, and the fourth centered on the DCE, exploring participants’ willingness to engage in grassroots-level work for an extended duration.

As presented in Table 4, within the category of identity characteristics, 48.89% participants were males, 48.76% participants were 31–40 age, 87.87% of participants were married, 79.55% were residents, 83.82% held a bachelor’s degree level education, and 53.48% held an intermediate title. Notably, the demographic characteristics of the respondents were markedly similar in both the online and offline modes.

**Table 4:** Findings of the study sample.

Category	Variables	Options	All (*N* = 445)		Online (*n*_1_ = 277)		Offline (*n*_2_ = 168)		*p*
			*n*	%	*n*	%	*n*	%	
Identity category topics	Gender	Male	222	49.89	140	50.54	81	48.21	0.067
		Female	223	50.11	137	49.46	85	50.60	
	Age	≤30	70	15.73	45	16.25	25	14.88	0.009
		31–40	218	48.76	126	45.49	91	54.17	
		41–50	100	22.47	68	24.55	32	19.05	
		>50	57	12.81	38	13.72	20	11.90	
	Marital status	Unmarried	49	11.01	35	12.64	14	8.33	0.452
		Married	391	87.87	237	85.56	154	91.67	
		Divorced	3	0.67	3	1.08	0	0.00	
		Widowed	2	0.45	2	0.72	0	0.00	
	Local registration	Yes	354	79.55	218	78.70	136	80.95	<0.001
		No	91	20.45	59	21.30	32	19.05	
	Academic degree	PhD	2	0.45	0	0.00	2	1.19	0.029
		Master	61	13.71	29	10.47	32	19.05	
		Bachelor	373	83.82	241	87.00	132	78.57	
		Specialized and below	9	2.02	7	2.53	2	1.19	
	Job title	Senior	57	12.81	35	12.64	22	13.10	0.001
		Deputy Senior	112	25.17	79	28.52	33	19.64	
		Intermediate	238	53.48	130	46.93	108	64.29	
		Junior	38	8.54	33	11.91	5	2.98	
	Form of employment	Career program	343	77.08	204	73.65	139	82.74	0.122
		Record system	40	8.99	31	11.19	9	5.36	
		Contract system	56	12.58	37	13.36	19	11.31	
		Unknown	6	1.35	5	1.81	1	0.60	
	Working years	≤10	154	34.61	90	32.49	64	38.10	0.367
		11–20	170	38.20	103	37.18	67	39.88	
		21–30	73	16.40	50	18.05	23	13.69	
		>30	48	10.79	34	12.27	14	8.33	
Sensitive topics	Annual income (million yuan)	≤6	25	5.62	25	9.03	0	0.00	0.061
		7–12	251	56.40	162	58.48	89	52.98	
		13–24	165	37.08	90	32.49	75	44.64	
		>24	4	0.90	0	0.00	4	2.38	
	Percentage of wages (%)	≤50	173	38.88	104	37.55	69	41.07	0.640
		51–75	216	48.54	143	51.62	73	43.45	
		>75	56	12.58	30	10.83	26	15.48	
	Percentage of desired income met (%)	≤50	102	22.92	70	25.27	32	19.05	<0.001
		51–75	149	33.48	98	35.38	51	30.36	
		>75	194	43.60	109	39.35	85	50.60	
Satisfaction rating topics	Difficulty of promotion	Difficult	206	46.29	151	54.51	55	32.74	0.024
		Average	227	51.01	119	42.96	108	64.29	
		Easy	12	2.70	7	2.53	5	2.98	
	Training opportunities	More	122	27.42	62	22.38	60	35.71	<0.001
		Average	252	56.63	160	57.76	92	54.76	
		Less	71	15.96	55	19.86	16	9.52	
	Interpersonal relations	Convivial	307	68.99	168	60.65	139	82.74	0.002
		Average	132	29.66	103	37.18	29	17.26	
		Not friendly	6	1.35	6	2.17	0	0.00	
	Recognition and respect from the public	Respectable	236	53.03	141	50.90	95	56.55	0.904
		Average	199	44.72	126	45.49	73	43.45	
		Not respected	10	2.25	10	3.61	0	0.00	
	Personal value embodiment	Reflective	193	43.37	113	40.79	80	47.62	0.215
		Average	236	53.03	152	54.87	84	50.00	
		Cannot show	16	3.60	12	4.33	4	2.38	
	Job satisfaction	Very satisfied	53	11.91	31	11.19	22	13.10	0.121
		Quite satisfied	212	47.64	124	44.77	88	52.38	
		Average	161	36.18	111	40.07	50	29.76	
		Quite dissatisfied	14	3.15	8	2.89	6	3.57	
		Very dissatisfied	4	0.90	3	1.08	1	0.60	
	Going to work in grassroots organizations	Very willing	25	5.62	16	5.78	9	5.36	0.840
		Willing	134	30.11	77	27.80	57	33.93	
		Fairly	142	31.91	99	35.74	43	25.60	
		Unwilling	130	29.21	77	27.80	53	31.55	
		Not sure	14	3.15	8	2.89	6	3.57	

Upon conducting a comparative analysis within the sensitivity category, it was observed that participants with an annual income of less than 60,000 yuan constituted 9.03% of the online survey participants, surpassing the 0% of the offline survey participants. Additionally, participants whose current income exceeded 75% of their desired income accounted for 39.35% of the online survey participants and less than the 50.60% of the offline survey participants.

In the comparative analysis of satisfaction evaluation category data, 54.51% of participants in the online survey perceived promotional opportunities as being challenging, 19.86% believed there were fewer training opportunities, 37.18% described their interpersonal relationships as being at an average level, and 40.07% reported having an average level of job satisfaction. These percentages were notably higher than those of participants in the offline survey.

### 3.3 Reasons for reluctance to go to primary health care organizations

**Figure 1:**
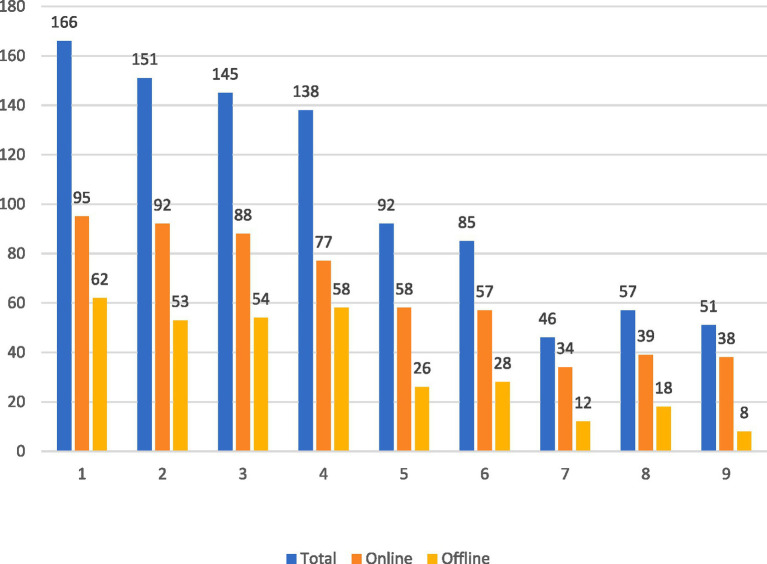
Distribution of the main reasons for not wanting to go to primary healthcare organizations. (1) Remote and inconvenient transportation. (2) Poor working environment and poor equipment. (3) Personal value not realized. (4) Low wages and treatment. (5) Low social status. (6) Opposition from family or friends. (7) High psychological pressure. (8) Difficulty in title promotion. (9) Others.

A total of 150 doctors expressed unwillingness, whereas 26 selected “not sure” in response to a question concerning their willingness to work in primary healthcare organizations from the online and offline survey, constituting a cumulative percentage of 39.55%. As illustrated in Figure 1, the primary concerns identified by the participants through the online survey comprised the following five items: (1) remoteness and inconvenient transportation; (2) poor working environment and basic equipment; (3) unrealized personal value; (4) low salary; and (5) low social status. Conversely, data collected through the offline survey revealed the following leading concerns: (1) remote and inconvenient transportation; (2) low salary; (3) unrealized personal value; (4) poor working environment and basic equipment; and (5) opposition from family or friends. The results derived from both research methods exhibited a substantial degree of consistency.

### 3.4 Heterogeneity and relative importance of preferences under the two survey methods

As shown in Table 5, all six job attributes exerted a significant degree of influence on physicians’ primary job choices. Both the online and offline survey results consistently highlighted that doctors assign the highest level of value to remuneration packages. A more in-depth analysis of the online findings revealed that when salaries were held as equal to the current level, doctors were 1.330 times more inclined to opt for a position in a village health office compared to instances where their current salary would be 20% lower (*p* < 0.001). Conversely, the offline survey results indicated that a 10% increase in the current salary raised the likelihood of a doctor choosing to work in a village health center by 1.095 times (*p* < 0.001) compared to scenarios in which their current salary would be reduced by 20%. Furthermore, the offline survey findings indicated a lack of preference heterogeneity among doctors regarding a 10% salary increase over their current level of salary (*p* = 0.898) and regarding the implementation of a filing system (*p* = 0.135).

**Table 5:** Attributes of traveling to primary care in the two research methods.

Properties	Preference	Online (*n*_1_ = 277)			Offline (*n*_2_ = 168)		
		Coefficient	SE	*p*	Coefficient	SE	*p*
Working organization	Village health center (ref)	0	—	—	0	—	—
	Township health center	0.082	0.124	0.006	0.481	0.132	<0.001
	Community health center	0.825	0.095	<0.001	0.995	0.116	<0.001
Remuneration package	20% lower than current salary (ref)	0	—	—	0	—	—
	The same as the current salary	0.951	0.111	<0.001	1.095	0.120	<0.001
	10% higher than current salary	1.330	0.13	<0.001	0.015	0.114	0.898
Title promotion	Consistent with hospital promotion years (ref)	0	—	—	0	—	—
	One-year advance promotion	0.092	0.094	0.030	0.350	0.097	<0.001
Types of employment	No establishment (ref)	0	—	—	0	—	—
	Filing system	0.319	0.110	0.005	−0.180	0.121	0.135
	With establishment	0.671	0.109	<0.001	0.648	0.118	<0.001
Weekly working hours	More than 60 h (ref)	0	—	—	0	—	—
	40–60 h	0.22	0.21	<0.001	0.029	0.113	<0.001
	40 h and below	0.232	0.102	0.024	0.331	0.120	0.011
Recognized and respected by residents	Not recognized and respected (ref)	0	—	—	0	—	—
	Average	0.21	0.13	<0.001	0.048	0.129	0.010
	More recognized and respected	0.82	0.14	<0.001	0.424	0.112	<0.001

**Figure 2:**
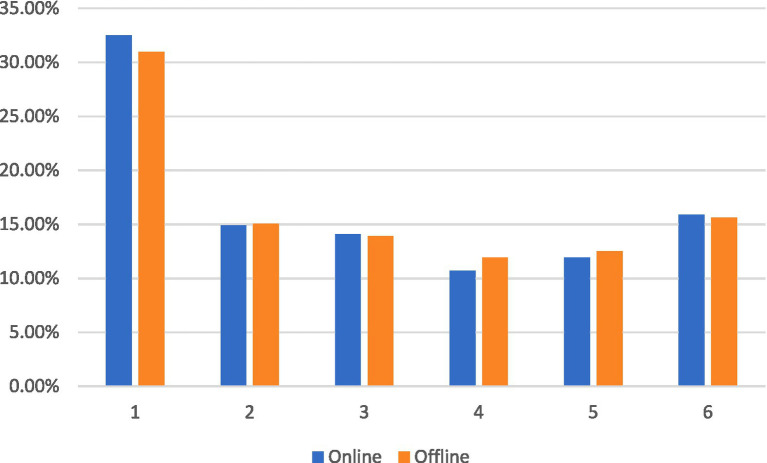
Percentage of relative importance of attributes under the two research methods. (1) Working organization. (2) Remuneration package. (3) Title promotion. (4) Types of employment. (5) Weekly working hours. (6) Recognized and respected by residents.

As illustrated in Figure 2, the relative importance of the attributes in both the online and offline survey results exhibited general consistency. The six attributes listed in descending order of importance were institution, mass recognition, salary, title, hours of work, and employment.

### 3.5 Scenarios forecasting analysis

The entire dataset was analyzed based on the estimation results of the hybrid logit model, as previously described. The choice probabilities for the three most crucial job attributes—working organization, being recognized, and respected by residents, and remuneration package—were simulated using a baseline scenario. This scenario was characterized as working in a village health office, being offered a salary 20% lower than participants’ current level of remuneration, the same anticipated duration for promotion, no staffing, over 60 working hours per week, and no recognition or respect from the local population.

In comparison to the established baseline, substituting the work institution with a township health center and community health center resulted in an increase of 7.06 and 13.92%, respectively, in terms of doctors’ choice probability. Similarly, replacing public recognition with an average level and a more recognized and respected level led to increases of 6.52 and 17.93%, respectively, in terms of doctors’ probability choice. When salary was replaced with levels equal to participants’ current salary and a salary 10% higher than their current salary, doctors’ choice probability increased substantially by 34.47 and 34.70%, respectively (see Table 6).

**Table 6:** Changes in choice probability of doctors in different work program scenarios (*N* = 445).

Scenarios No.	Working Organization	Remuneration package	Title promotion	Types of employment	Weekly working hours	Recognized and respected by residents	Probabilistic change	Effect 95% CI	
Ref	1	1	2	2	2	1	—	—	—
①	2	1	2	2	2	1	7.06%	8.55%	5.57%
②	3	1	2	2	2	1	13.92%	15.53%	12.30%
③	1	1	2	2	2	2	6.52%	7.89%	5.15%
④	1	1	2	2	2	3	17.93%	20.69%	15.18%
⑤	1	2	2	2	2	1	34.47%	36.23%	32.70%
⑥	1	3	2	2	2	1	34.70%	37.23%	32.16%

## 4 Discussion

While numerous contemporary studies have conducted DCE surveys examining physicians’ preferences regarding primary care work, there is a conspicuous scarcity of research focusing on the primary work preferences of seasoned physicians in secondary or county-level general hospitals in China. Additionally, the gap in China’s grassroots health manpower remains substantial, with an even more pronounced shortage of high-quality doctors. There is a notable dearth of research exploring the influence of survey methods on the implementation of DCE among physicians. This study aimed to establish a foundational framework for selecting survey methodologies suitable for conducting DCEs among physicians. Furthermore, this study seeks to identify and enable the development of more precise incentive programs with the goal of enticing experienced physicians to engage in grassroots-level healthcare.

In the examination of the cost and questionnaire quality associated with the two survey methods, online and offline, it became evident that online surveys offer several distinct advantages, including cost and labor efficiency, as well as the capacity to engage many participants across a diverse range of districts and institutions. However, online surveys also exhibit lower questionnaire efficiency than offline surveys. In contrast, offline surveys have contrasting characteristics. This observation aligns with the findings of other studies that have employed questionnaire surveys (Riva et al., 2003; Tolstikova and Chartier, 2009). While DCE questionnaires differ from general-scale questionnaires, offline surveys provide the opportunity to mitigate respondents’ misunderstandings and elicit real-time feedback and corrections, thereby enhancing the validity of offline questionnaires. Meanwhile, given that the respondents in this survey were doctors, a highly educated group with a heightened understanding and sense of responsibility about the responses provided to DCE questionnaires, this elevated the validity of the questionnaire across both surveys. In the underlying descriptive statistics, noteworthy differences emerged between the results obtained from the two survey methods, particularly in the sensitive topics and satisfaction evaluation categories. Regarding the sensitive topics category, the outcomes from online surveys consistently reflected lower reported incomes, which aligns with the findings of other researchers (Wang et al., 2019). Within the satisfaction evaluation category, the overall results obtained through the online surveys indicated lower levels of satisfaction across several aspects, a trend that was more consistent with the Sixth Health Services Statistics Survey of the China Health Services Survey. Drawing on the theory of social appropriateness (Marlowe and Gottesman, 1964), it has been suggested that online surveys alleviate the social pressure placed on the doctors being interviewed, enabling more genuine rather than socially expected responses. Consequently, the authenticity of the online surveys was presumed to be higher.

Furthermore, 39.55% of the interviewed doctors expressed reluctance or uncertainty regarding their inclination to work in primary care organizations. Drawing on the principles of prospect theory, individuals making choices amid uncertainty may deviate from expected utility theory outcomes by prioritizing loss minimization over gain maximization (Constantinople et al., 2019). The physicians interviewed in this study were obtained exclusively from secondary or county-level general hospitals in Eastern China, wherein they benefited from a comprehensive working environment superior to that provided by community health centers, township health centers, and village health offices. Consequently, their reluctance to consider positions at the grassroots level is rooted in the advantageous nature of their current professional environments. This is in line with the current shortage of primary care physicians (Islek and Sahin, 2023).

Using a constructed mixed logit model, this study examined the heterogeneity and relative importance of doctors’ primary job preferences. Both the online and offline surveys consistently indicated that work organization, remuneration, and public recognition stand out as the most crucial job attributes influencing doctors’ decisions to work at the grassroots level. This aligns with the findings previously reported by other researchers (Rao et al., 2013; Park and Ko, 2016; Saran et al., 2020; Law et al., 2021).

First, variations in the available resources across primary care organizations in different locations contributed to the observed differences. Compared with other organizational types, community health resources encompass more advanced medical equipment, pharmaceuticals, human resources, and opportunities for career development. This abundance of available resources enables physicians to deliver high-quality health services and advance their professional development. Second, fair remuneration is regarded as compensation for the considerable effort invested by doctors, particularly in the context of the relatively low-income levels that doctors in China receive. Previous research has indicated that doctors are more inclined to work for organizations that offer fair compensation (Abdel-All et al., 2019). Given the multifaceted nature of doctors’ roles in township health centers and village health offices, which include the provision of basic medical services, health management, and basic public health services, higher compensation is anticipated to reflect the elevated demands and responsibilities associated with their roles. Finally, drawing on the principles of Maslow’s needs theory (Poh et al., 2022). When doctors’ talents and potential are acknowledged by the broader population, this becomes a motivating factor behind their decision to work in primary healthcare institutions. This choice was ranked lower than that of healthcare workers in other developed countries (Manahan et al., 2009), but higher than that of healthcare workers in other developing countries (Manda et al., 2023). Additionally, doctors expressed the lowest level of preference for bianzhi, a finding that is not entirely consistent with our anticipated results and with some existing research (Liu et al., 2022). This discrepancy may be attributable to the recent de-bianzhi reforms implemented in China. Our outcome suggests that this reform has achieved certain notable successes thus far.

Scenarios prediction analysis revealed that the highest level of probability of doctors choosing to work in primary care organizations occurred under the combination of a “village health office + 20% increase in salary + one-year advance promotion + filing system + 40–60 h of work per week + residents’ recognition and respect in general.” This finding underscores the fact that the factor of enhanced remuneration has the most significant impact on increasing doctors’ willingness to work in primary care organizations. This suggests that physicians tend to choose job options (e.g., higher pay, promotion opportunities) where they can avoid losses, which is consistent with the behavioral model in prospect theory (Stewart et al., 2003). The results of related studies also highlight the substantial income gap that exists between primary care doctors and their hospital-based counterparts (Liu et al., 2022). In conjunction with other research findings (Soekhai et al., 2019; Bao and Huang, 2021; Bao et al., 2023), this is one of the primary reasons for the current shortage of primary care physicians. Therefore, improving remuneration is a crucial step toward enhancing the appeal of Chinese primary health organizations to high-quality doctors.

## 5 Implications

In summary, this study makes several recommendations. First, regarding methodology, it is recommended that future applications of DCE utilize online research for groups with high educational levels, such as doctors, as this will not only reduce the costs for both investigators and respondents but also enhance the reliability and validity of the data. Regarding incentive policies for health talents, it is suggested that the health sector increase salary subsidies for primary care doctors and improve the infrastructure development and medical resource allocation of primary care institutions to enhance the preference for primary care among doctors. Additionally, in response to the current large gap in health manpower at the grassroots level in China, policymakers in the health sector can enhance doctors’ social respect and sense of self-realization by recognizing outstanding doctors and providing honorary titles, thereby increasing their willingness to work at the grassroots level. Finally, it is suggested that policy makers consider implementing more flexible working arrangements at the grassroots level, such as flexible working hours and shift work systems, to reduce the workload of doctors and increase their job satisfaction.

## 6 Limitations

First, DCE are primarily validations of short-term experiments. In the future, they can be combined with Markov modeling for medium- and long-term predictions and planning regarding healthcare workers’ work preferences. Second, the DCE questionnaire designed for this study did not include other potential incentives or job characteristics that could significantly influence physicians’ job preferences. Finally, regarding the congruence between the results of the questionnaires and real-world scenarios, although the superior authenticity of the online survey method over offline survey was ascertained, the alignment between the outcomes of questionnaires and the actual situation discussed in this study necessitates further validation.

## 7 Conclusion

This study specifically addresses the effect of DCE survey method on physicians’ choice of job preferences, thereby filling a research gap on the preferences of physicians in secondary hospitals for choosing primary jobs. It revealed that online surveys offer the advantage of cost savings, and that the questionnaire validity rate was higher for both survey methods. Consequently, it is advisable to employ online questionnaires to conduct DCE surveys of respondents with higher education levels and better comprehension abilities. Simultaneously, through the construction of a mixed logit model, both survey methods employed indicated that doctors in secondary- or county-level general hospitals prioritized concerns related to work organization, remuneration, and public recognition. Specifically, the optimal scenario identified for enhancing the probability of doctors choosing to work in primary healthcare institutions can be described as “village health office + 20% increase in salary + one-year advance promotion + filing system +40–60 h of work per week + residents’ recognition and respect in general.” This underscores the clear importance of improving remuneration packages and provides valuable references and insights for future surveys and policy formulations in related contexts. It is recommended that health policymakers target doctors by increasing their salaries, rewarding them with honors, and allowing them to have flexible working hours at the grassroots level.

## Data Availability

The raw data supporting the conclusions of this article will be made available by the authors, without undue reservation.
